# Hydrodynamic reception in the Australian water rat, *Hydromys chrysogaster*

**DOI:** 10.1007/s00359-020-01416-8

**Published:** 2020-04-18

**Authors:** Wolf Hanke, Sabine Meyer, Horst Bleckmann, Guido Dehnhardt

**Affiliations:** 1 Sensory and Cognitive Ecology, Institute for Biosciences, University of Rostock, Albert-Einstein-Strasse 3, 18059 Rostock, Germany; 2 Institute for Zoology, University of Bonn, Poppelsdorfer Schloß, 53115 Bonn, Germany

**Keywords:** Hydrodynamic reception, Rodent, *Hydromys chrysogaster*, Predation, Vibrissal system

## Abstract

The Australian water rat, *Hydromys chrysogaster*, preys on a wide variety of aquatic and semiaquatic arthropods and vertebrates, including fish. A frequently observed predatory strategy of *Hydromys* is sitting in wait at the water's edge with parts of its vibrissae submersed. Here we show that *Hydromys* can detect water motions with its whiskers. Behavioural thresholds range from 1.0 to 9.4 mm s^−1^ water velocity, based on maximal horizontal water velocity in the area covered by the whiskers. This high sensitivity to water motions would enable *Hydromys* to detect fishes passing by. No responses to surface waves generated by a vibrating rod and resembling the surface waves caused by struggling insects were found.

**Electronic supplementary material:**

The online version of this article (10.1007/s00359-020-01416-8) contains supplementary material, which is available to authorized users.

## Introduction

Many aquatic and semiaquatic mammals use their vibrissae to obtain information by directly touching and investigating unknown objects (Dehnhardt 1990, 1994; Dehnhardt and Kaminski 1995; Dehnhardt and Dücker 1996; Dehnhardt et al. 1998b; Catania et al. 2008). Harbour seals (*Phoca vitulina*) use their vibrissae to detect minute subsurface water motions (Dehnhardt et al. 1998a). Harbour seals (Dehnhardt et al. 2001; Wieskotten et al. 2010) and California sea lions (Gläser et al. 2011) can even follow the hydrodynamic trail left behind by a moving object. Similar to pinnipeds, many aquatic and semiaquatic mammals associated with fresh water habitats have vibrissae which they might also use to detect prey-generated water motions. For instance, the African water rat *Colomys goslingi*, which hunts tadpoles and fish at night in shallow rivers, often sits at the waterside with its vibrissae immersed. Fish eating cats (*Prionailurus viverrinus*) show a similar behaviour (Seidensticker and Lumpkin 1991; Heydon and Ghaffar 1997), and otter civets (*Cynogale bennettii*) have been observed to hunt in the shallows of a river with their snout, which bears prominent vibrissae, positioned just above or below the water surface. American water shrews (*Sorex palustris*) attack the source of brief (75 ms) pulsed water movements, while experiments with live prey fish gave no indication that they pursue hydrodynamic trails (Catania et al. 2008).

**Fig. 1:**
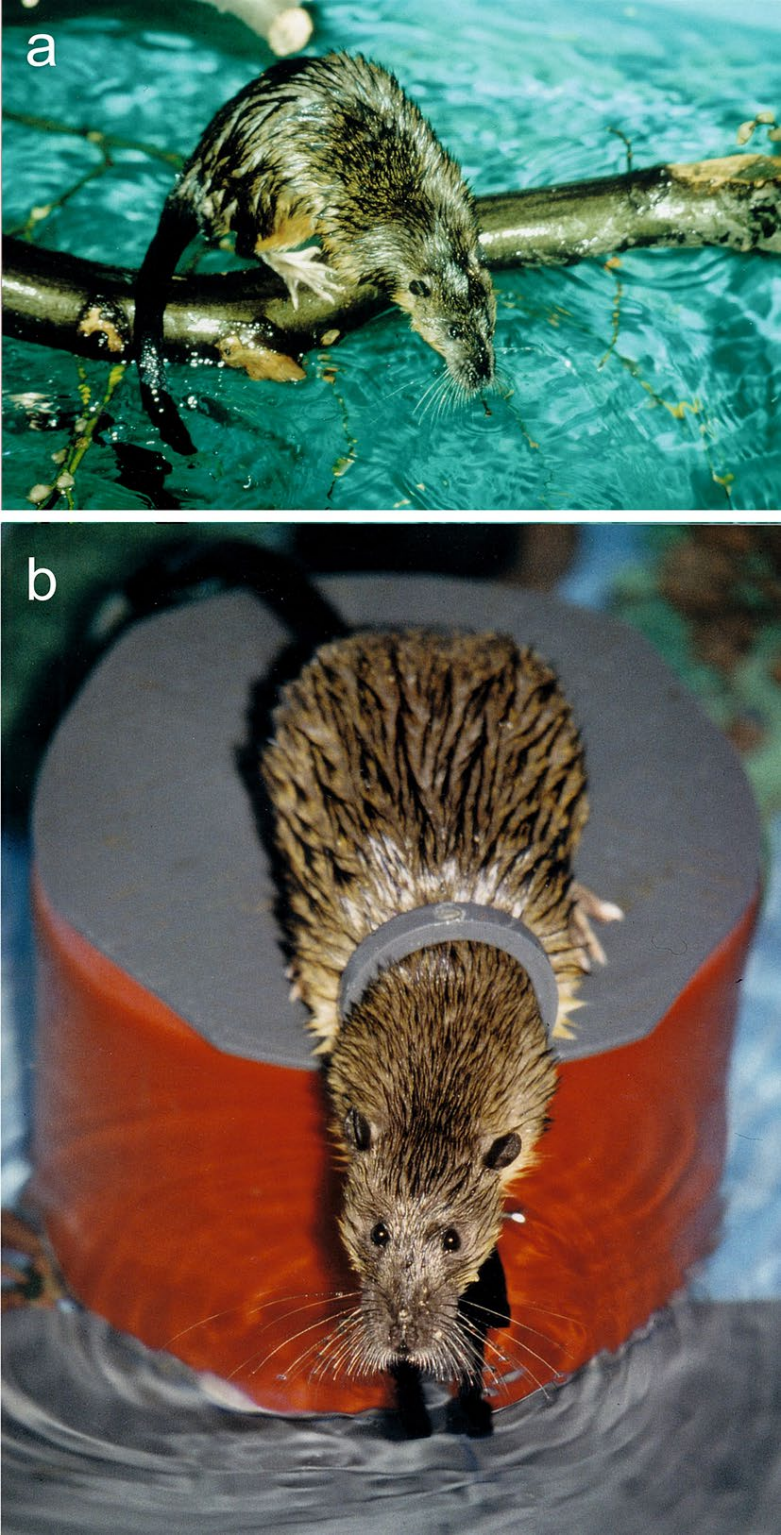
The Australian water rat, *Hydromys chrysogaster*, performing its sit-and-wait behaviour with immersed vibrissae; **a** spontaneously in a near-natural environment, **b** induced to station in the experimental setup

The Australian water rat *Hydromys chrysogaster* (Fig. 1) inhabits river banks, lakes and sea shores (McNally 1960). *Hydromys chrysogaster* is an opportunistic predator which feeds on aquatic and semiaquatic insects, spiders, crustaceans, mussels, water birds and fish (McNally 1960; Woollard et al. 1978). Like many other fish hunting mammals, *H. chrysogaster* has well developed vibrissae which are densely innervated and possess a variety of morphologically different nerve endings (Dehnhardt et al. 1999). Based on results from confocal microscopy in *Rattus norvegicus* (Ebara et al. 2002), a fellow member of the rodent family Muridae, it may be assumed that at least eight different types of nerve endings are present also in *Hydromys*. However, vibrissal follicle-sinus complexes of *H. chrysogaster* are larger by about 60% and are more densely innervated by a factor of at least 2.5 than the vibrissae of terrestrial rodents like *R. norvegicus* (Dehnhardt et al. 1999).

While in ambush, *H. chrysogaster* spreads out its vibrissae so that their tips are submerged (Fig. 1a). This behaviour, together with the observation that *H. chrysogaster* closes its eyes while swimming and diving, suggests that Australian water rats use their vibrissae for the detection of prey-generated water motions. Since *Hydromys chrysogaster* readily displays its natural sit-and-wait-behaviour under laboratory conditions (Fig. 1b), we tested this hypothesis in two psychophysical experiments with two different kinds of hydrodynamic stimuli. Hydrodynamic stimuli 1 (used in experiment 1) consisted of surface waves to mimic an insect struggling at the water surface, while hydrodynamic stimuli 2 (used in experiment 2) were generated by subsurface water motions to mimic a prey item below, but close to, the water surface. Hydrodynamic stimuli 2 involved subsurface and surface water flow as well as surface waves.

## Material and methods

### Animals, housing and experimental pool

Two female and one male Australian water rat, *H. chrysogaster* (Muridae), were used for the experiments. Animals were from the same litter and were bred and raised at the University of Bielefeld, Germany. In the following, the animals are designated with “Male 1”, “Female 1” and “Female 2”. At the beginning of the study, the water rats were about 6 months old. Body masses were between 800 and 1200 g.

All three animals completed experiment 1. However, Female 2 died prior to experiment 2. Therefore, only Male 1 and Female 1 completed the second experiment.

Animals were individually held in cages sized approximately 140 cm × 80 cm, each of which had a land and a water part (water depth 45–50 cm). They were fed once a day with 60 g of shrimps, fish, mollusks, or cat food. Half of the food was given during experimental sessions and training.

**Fig. 2:**
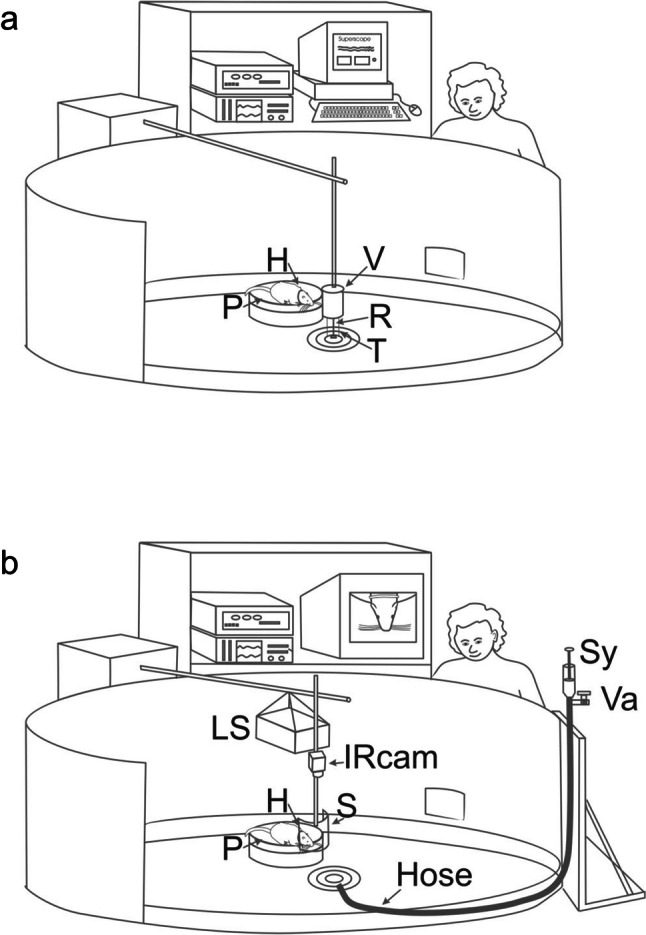
Experimental setup for the application of stimuli 1 (**a**) and stimuli 2 (**b**). In both setups, the animal rested on a platform (P) 1 cm above the water surface and placed its head through a hoop (H, c.f. Fig. 1b) with the vibrissae immersed. **a** Stimuli 1: surface waves were generated with a rod (R) mounted to a vibrator (V). A plastic tube (T) that ended close to the water surface prevented the animal from seeing or touching the rod. **b** Stimuli 2: complex hydrodynamic stimuli were generated by letting water flow out of a hose (Hose) that ended 14 cm below the water surface. The stimulus was generated by the hydrostatic pressure of the water that had been sucked above the surface with a syringe (Sy), then released by opening a valve (Va). A hollow shield (S) prevented the animal from moving too close to the stimulus origin. An infrared camera (IRCam) recorded the animal and displayed it on a monitor. A loudspeaker (LS) above the animal delivered pink noise to mask acoustic cues

Experiments were conducted in a separate circular indoor pool (diameter 1.5 m, height 35 cm) (Fig. 2a, b). The perimeter of the pool was enclosed by PVC plates to prevent the animals from escaping. Water depth was 24.5 cm in all experiments. An oval experimental platform was mounted in the middle of the pool 1 cm above the water surface. A stationing hoop (inner diameter 4.7 for the females and 5.4 cm for the male) was attached to the experimental platform; the animal was trained to place its head through the hoop to achieve a reproducible position (Figs. 1b, 2a, b). One water rat at a time was allowed to enter the pool for an experimental session and to haul out on the experimental platform. Water temperature in the pool and in the cages was 20 to 25 °C.

### Hydrodynamic stimuli 1 (pure surface waves)

Water surface waves were produced with a rod mounted to a Ling mini shaker (Ling Dynamic Systems, Royston, UK, model 201/PA 25E) (Fig. 2a). Three different rods of diameters 0.5 cm, 2 cm and 3 cm were used to generate surface waves of different amplitude. Rods were 19.5 cm long and were immersed 2 mm into the water. Surface waves were generated by oscillating the rod vertically with the Ling mini shaker. The holder of the shaker rested on a separate platform to ensure that no unwanted vibrations were transmitted indirectly to the animals via the ground. The electronic signals driving the shaker were synthesized with a computer (Apple Power Macintosh IIci), output via a 14-bit DA-converter at a conversion rate of 20 kHz (MacAdios II, GW-Instruments, Somerville, MA, sampling rate 10 kHz), and power amplified (Ling Dynamic Systems, model PA25E). To rule out that the water rats could sense the vibrating rod by directly touching it with the vibrissae or by visual cues, the portion of the rod that was above the water surface was encased in a plastic tube (outer diameter 6 cm). With the software Superscope II 1.44 (GW-Instruments, Somerville, MA) and a custom-made macro (M. Kettler, University of Bonn), frequency, duration and amplitude of the generated surface wave stimuli could be varied at will. In this manner, single-frequency sine wave stimuli (10, 20, 30, 40 and 50 Hz) and broadband noise stimuli (bandwidth 0–50 Hz, 0–100 Hz, and 0–200 Hz) were created. Broadband stimuli were used since they simulate, unlike single-frequency stimuli, natural, prey-generated surface waves (Bleckmann 1985a, b). Stimulus duration varied between 1 and 3 s. To avoid stimulus onset and offset artefacts, rise and fall times of surface wave stimuli were adjusted to 250 ms.

Surface waves were recorded with a receiver electrode (a chlorided silver wire of 200 µm diameter) that was partly immersed into the water. The receiver electrode was placed at the location where the water rat would position its snout during an experimental session. A 10 cm long silver wire, submerged into the water, served as a reference electrode. The method of wave measurement was based on the principle that the electrical resistance between a fully submerged reference electrode and a partly immersed receiver electrode depends on the immersion depth of the latter. Resistance was measured with a Wheatstone bridge circuit (custom made, University of Bielefeld). The Wheatstone bridge circuit outputs a voltage (measured in mV) that is transformed to surface wave amplitude based on the calibration curve for the system. To plot the calibration curve, the immersion depth of the receiver electrode was changed with a micromanipulator. During surface wave measurements no animals were present.

**Fig. 3:**
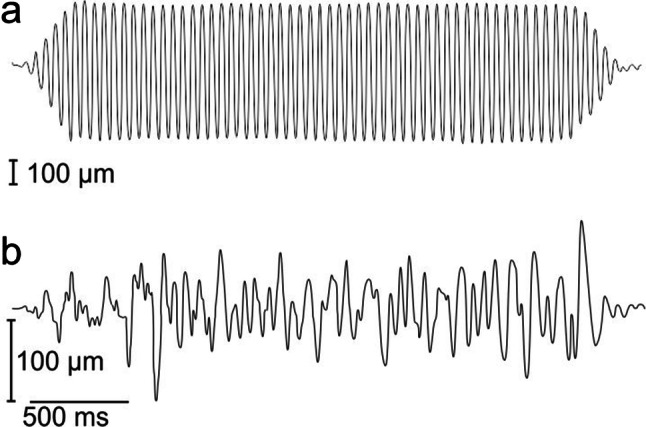
Examples of water surface waves generated with a vibrating rod (stimuli 1, experiment 1, detection of pure surface waves), quantified using an immersed wire connected to a Wheatstone bridge. **a** 20 Hz sine wave with rise and fall times of 250 ms. **b** broad band surface wave stimulus

The results of surface wave quantification of stimuli 1 are summarized in Table 1. Figure 3 shows an example of a single-frequency surface wave (a) and a large bandwidth surface wave stimulus (b), recorded with a Wheatstone bridge circuit. The single-frequency surface waves used in the behavioural experiments had mean peak-to-peak displacement amplitudes (*n* = 3) of 910 µm (10 Hz), 690 µm (20 Hz), and 100 µm (40 Hz), respectively. For small or large bandwidth stimuli, maximal mean peak-to-peak amplitudes were 115 μm (0–50 Hz), 165 μm (0–100 Hz), and 210 μm (0–200 Hz), respectively.

**Table 1:** Measured surface wave amplitudes for stimuli 1 (used in experiment 1); mean values of three stimulus repetitions

Frequency in Hz	Mean p–p amplitude (µm)
Single-frequency stimuli	
10	910
20	690
40	100
Broadband stimuli	
0–50	115
0–100	165
0–200	210

Amplitudes were averaged over the stimulus duration (excluding onset and offset times)

### Hydrodynamic stimuli 2 (complex hydrodynamic stimuli)

Complex hydrodynamic stimuli, including subsurface and surface water motions, were generated with a hose (inner diameter 1 cm) that ended 14 cm below the water surface, 20 cm in front of the platform. The opening of the hose pointed towards the platform such that the angle between the hose and the water surface was 70°. The hose followed the bottom of the experimental tank and finally left the water to end 80 cm above the water surface (Fig. 2b). By means of a syringe, a defined amount of water could be sucked into the hose and above the water level, thus generating hydrostatic pressure. On opening a valve (No. 976, Gardena, Ulm, Germany), the water pressure was released and generated subsurface and surface water motions whose strength was a function of the hydrostatic pressure, i.e. of the height of the water column in the hose relative to the water surface in the experimental tank. The water velocity at the lower end of the hose could be varied at will by varying the height of the water column. The height of the water column could be adjusted with an accuracy of 0.5 cm.

A custom-made particle tracking device was used to visualize and measure water movements. In particle image velocimetry and particle tracking velocimetry, water velocity is quantified by adding small buoyant particles to the water, illuminating a plane in the water using a fanned-out or moving laser beam, and recording a sequence of images with a camera from an angle perpendicular to the illuminated plane. Here, a horizontal plane 5 mm below the water surface was illuminated with an array of ten diode lasers (< 5 mW each). The beam of each diode laser was passed through a glass rod that served as a cylinder lens and spread the beam in one direction, thus generating a light sheet approximately 1 mm thick and 100 mm wide. To visualize water movements, neutrally buoyant seeding particles (Vestosint 1101, Hüls AG, Marl, Germany; median diameter 100 µm) were seeded into the water. Pictures were taken from below with submerged CCD camera modules (0.2 lx, Conrad Electronic, Hirschau, Germany) that were equipped with VT objectives (8 mm, *f* = 1.2). Pictures were recorded on VCR (Panasonic NV-F70 HQ) at the time of the experiments. Videos were digitized post measurement using an LG V4745 video recorder whose signal was looped through a JVC SR-VS20 video recorder to obtain a Digital Video (DV) signal. Videos were processed using MatLab R2019b (www.mathworks.com) and Virtual Dub 1.10.4 (www.virtualdub.org), and particle velocities were measured using ImageJ 1.52a (https://www.imagej/nih.gov/ij). In this way, water velocities were related to the water column height that was used to generate stimuli 2. However, not only the particles in the laser light sheet 5 mm below the water surface, but also particles floating on the water surface were illuminated, the latter by reflected laser light with lower intensity. While this effect prevented the videos from being evaluated with correlation techniques as they are common in particle image velocimetry (PIV), it also offered the opportunity to measure water velocities in two different planes, namely the laser light sheet 5 mm below the water surface as well as the water surface itself. For details of the evaluation procedure and example movies of the water flow, see supplementary materials. Results are summarized in Table 2.

**Table 2:** Results of experiment 2 along with surface wave parameters, flow parameters measured using particle tracking (see Supplementary Materials for details of methods and examples), and number of trials

Water column height (cm)	Surface wave maximal amplitudes (μm)	Surface wave mean amplitude (μm)	Time of arrival of surface waves	Surface water velocity (mm s^−1^)	Sub-surface water velocity (mm s^−1^)	%Correct male 1	Number of trials male 1	Hits male 1	Misses male 1	False Alarms male 1	%Correct female 1	Number of trials female 1	Hits female 1	Misses female 1	False Alarms female 1
5				0.21^a^							30	20	6	14	18.9% of 254 blank trials
10				0.97 ± 0.29		29	24	7	17	15.9% of 270 blank trials	72	21	15	6	
15	65 ± 32	15 ± 5	1394 ± 87	4.64 ± 1.23											
20	201 ± 73	46 ± 13	1061 ± 88	5.78 ± 0.48		29	24	7	17		75	20	15	5	
25	435 ± 143	95 ± 26	974 ± 67	12.42 ± 3.30		48	23	11	12						
30	772 ± 211	151 ± 30	886 ± 78	21.64 ± 5.09	32.77 ± 2.72	67	61	41	20		88	52	46	6	
40	1370 ± 249	241 ± 39	725 ± 38		54.63 ± 7.21	99	109	108	1		89	92	82	10	
76. 5	6125 ± 1329	1000 ± 153	629 ± 33		142.96 ± 17.05	100	43	43	0	17% of 43 blank trials	95	41	39	2	9% of 41 blank trials

^a^Designates an extrapolated value

**Fig. 4:**
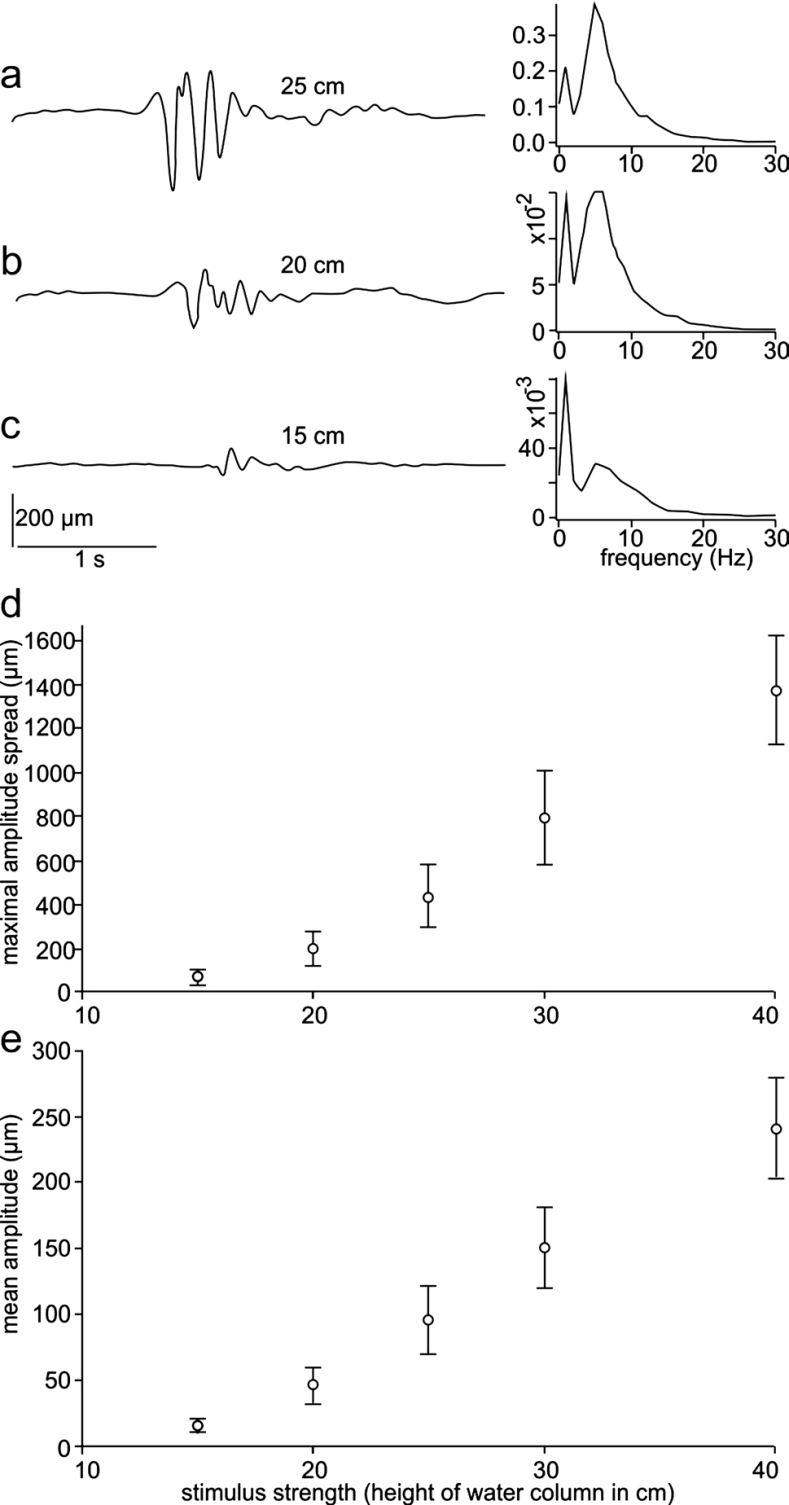
Surface waves that were part of the complex hydrodynamic stimuli generated with a hose (stimuli 2, experiment 2, detection of complex hydrodynamic stimuli). **a**–**c** Examples of typical surface waves (left) and their frequency spectra (right). Spectra were averaged over 20 repetitions. Stimuli were generated with water column heights of 25 cm, 20 cm, and 15 cm, respectively. **d** Maximal amplitudes for water column heights from 15 to 40 cm. **e** Mean amplitudes for water column heights from 15 to 40 cm

Hydrodynamic stimuli 2 included also surface waves, i.e. vertical surface movement. Figure 4a–c shows the typical time course (left) and averaged frequency spectra (right) of surface waves (*n* = 20) generated by the water columns. Peak-to-peak displacement amplitudes of the surface waves, measured with the Wheatstone bridge circuit, were 6125 ± 1329 µm (height of water column 76.5 cm), 1370 ± 249 µm (40 cm), 435 ± 143 µm (25 cm), and 65 ± 32 µm (15 cm). Surface waves reached the measurement location after 629 ± 33 ms (76.5 cm), 724.5 ± 38 ms (40 cm), 974 ± 67 ms (25 cm), 1394 ± 87 (15 cm), respectively. Surface waves generated by the 5 cm and 10 cm water column were smaller than that and were below the resolution of our measurement device. Stimulus parameters for stimuli 2 (experiment 2) are summarized in Table 2, along with the behavioural performance data.

### Animal training

Animals were trained using operant conditioning and positive reinforcement, taking advantage of species-typical behaviour. Once they had settled on the experimental platform (Figs. 1b, 2), the animals spontaneously scanned the water surface with their vibrissae using head movements. If the animal placed its head through the stationing hoop, food was presented with a tweezer. Once the animals had learned to place their head through the hoop, they had to remain in the hoop for a specified time that was gradually increased to 10 s. After this time the experimenter gave a short high-frequency signal with a training whistle (secondary reinforcer). After the whistle signal, food was offered immediately (primary reinforcer).

In a second step, animals were trained to a go/no-go response paradigm: they had to leave the hoop (go response) when a hydrodynamic stimulus was presented manually with a rod or to stay in the hoop for at least 10 s (no-go-response) if no stimulus was presented. Correct behaviour was followed by the secondary and primary reinforcer. Initially, the animals received additional cues in the form of a verbal command or direct touch of the whiskers with the rod in case a go response was correct. All animals learned the go/no-go response quickly and maintained it after the additional cue was removed. In the initial experiments, visual or auditory cues were not masked, resulting in multimodal stimuli.

### Experimental procedure

Animals were experimentally naive at the beginning of the study, and performed reliably in the go/no-go response paradigm with multimodal stimuli when testing began. Each experimental animal took part in two experimental sessions per day, each session consisted of up to 20 trials. Hydrodynamic stimuli were given only in about half of the trials. Trials with and without (blank trials) a hydrodynamic stimulus were performed in pseudorandom order (Gellermann 1933). At most three rewarded trials or three blank trials were presented in a row. On trials where a stimulus was given, it was delivered after the water rat had stationed in the hoop for at least 2 s (the time course of each trial was controlled with a digital stopwatch). Behavioural responses in trials where a stimulus was presented were scored as a “hit” (correct response) if the water rat left the hoop within 4 s after the valve had been opened (i.e. 6 s total stationing time in the hoop), and as a “miss” else. Behavioural responses in trials where no stimulus was presented were scored as a “correct rejection” if the water rat stayed in the hoop for 10 s total, and as a "false alarm" else. Hits and correct rejections were reinforced with the high-frequency whistle signal that had been established as a secondary reinforcer. Immediately after the whistle signal, the experimental animal was rewarded with food. Misses and false alarms were not reinforced. At the beginning of each experimental session, three warm-up trials were performed. These trials are not included in the data set. We started data collection only if at least two out of the three warm-up trials were successful. Otherwise, more warm-up trials were performed or the session was terminated.

To exclude visual cues, experiments were performed under infrared illumination. The water rats were filmed with an IR camera (CCD camera module No. 192589, Conrad Electronic, Hirschau, Germany) whose output signal was displayed on a TV monitor. To avoid any unwanted illumination, the monitor screen was shielded with black paper. The experimenter could, however, view the monitor through a small slit in the paper shield. To deliver a food reward, a light bulb was switched on.

To mask acoustic cues, pink noise (0–20 kHz; sound pressure level 86.7 dB re 20 μPa) was generated with a loudspeaker positioned above the experimental platform. The masker noise was not presented continuously throughout the session, but on each trial. The sound pressure level was measured near the stationing hoop with a hydrophone (Bruel & Kjaer 8103) and a charge amplifier (Bruel & Kjaer 2635).

Experiment 1, i.e. stimulation with pure surface waves (hydrodynamic stimuli 1), was conducted prior to experiment 2, i.e. stimulation with complex hydrodynamic stimuli (hydrodynamic stimuli 2). Both experiments were conducted to test whether the experimental animals responded to the respective type of stimulus at all. If this was the case, we determined the behavioural thresholds of the experimental animal.

Threshold measurements, as it turned out, were possible only with hydrodynamic stimuli 2. During threshold measurements, the order of stimulus presentation followed the method of constant stimuli (Gescheider 1976). In each session, water column heights of 10 cm, 20 cm, 30 cm, and 40 cm were presented in pseudorandom order, pseudorandomly mixed with an equal number of blank trials where no stimulus was presented. In addition, a 25 cm condition was used for Male 1, and a 5 cm condition was used for Female 1. The strongest stimulus with a water column height of 76.5 cm was only used in the preceding stage of the study, and was not presented during threshold measurements.

## Results

### Experiment 1: responses to stimuli 1 (pure surface waves)

None of the three subjects showed a spontaneous response to single-frequency stimuli or to small or large bandwidth surface waves. To facilitate learning, an additional stimulus was introduced that indicated the presence of a hyrodynamic stimulus, with the intention to gradually remove the additional stimulus in the progress of the experiment (“fading” as in Terrace 1963) so that the hydrodynamic stimulus would gain control over the animal's response. Additional stimuli were a verbal command if single-frequency surface waves were applied, or touching the animal with a rod if large bandwidth stimuli were applied. However, while all animals quickly learned to apply the go/no-go response scheme in the presence of the additional stimulus, they did not respond to the hydrodynamic stimulus alone once the additional stimulus was removed. After at least 4 weeks of training with no improvement, training to stimuli 1 was terminated.

### Experiment 2: responses to stimuli 2 (complex hydrodynamic stimuli including horizontal flow and vertical surface waves)

In the first set of experiments with stimuli 2, we presented water motions generated with a 76.5 cm water column (76.5-cm-stimulus). In the area covered by the animal’s vibrissae maximal water velocity was 143 mm s^−1^, measured 5 mm below the water surface.

Both experimental animals responded spontaneously to this stimulus. While Male 1 showed a strong avoidance reaction in the very first trial, Female 1 spontaneously moved its head in the direction of the stimulus source. During four consecutive days, the two animals tested in this condition responded strongly in nearly all of the trials in which a stimulus was presented (Male 1, 100% of 43 trials; Female 1, 95% of 41 trials). False alarm rates were 17% (Male 1, 43 blank trials) and 9% (Female 1, 41 blank trials).

**Fig. 5:**
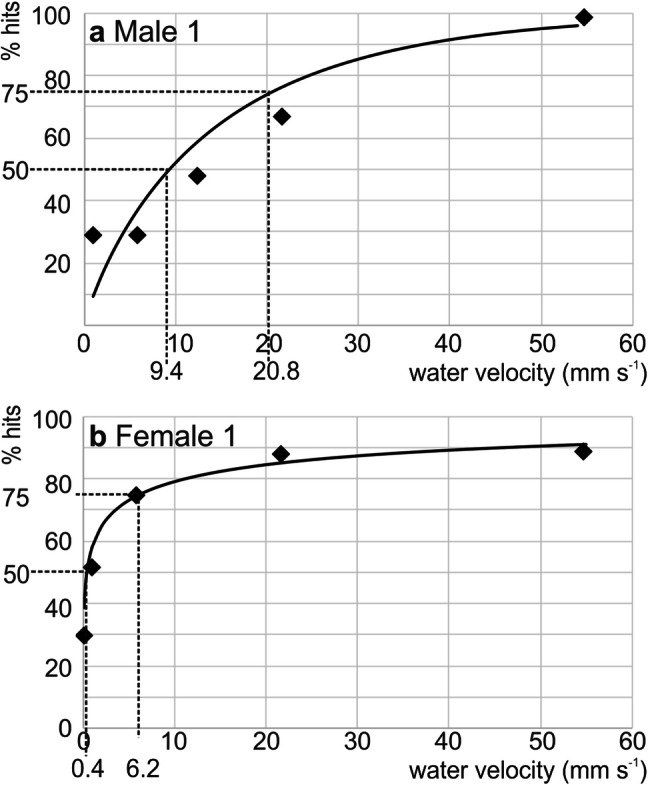
**a, b** Psychometric functions of the two water rats that participated in experiment 2. Percentage of correct responses (*y* axis) to the complex stimuli as function of stimulus amplitude (maximal water velocity within the reaction time, measured using floating tracer particles). Data are fitted with a Weibull function. The horizontal dashed lines mark 50% and 75% correct responses, respectively. The vertical dashed lines indicate the water velocity at which 50% or 75% correct responses were obtained. **a** The male animal (male 1), **b** the female animal (female 1)

In a second set of experiments with stimuli 2, psychometric functions for both animals were assessed using water column heights from 5 to 40 cm (see methods and Table 2). Psychometric functions are shown in Fig. 5. To plot these functions, water velocity measured using the floating particles rather than surface wave amplitude measured using the Wheatstone bridge was taken as the presumably more relevant parameter of the hydrodynamic stimulus, taking into account that stimuli 1, which consisted of pure surface waves, had failed to elicit any response in the three animals used in experiment 1. Both experimental animals responded reliably to water velocities of 55 mm s^−1^. Hit rate was 99% (Male 1) and 89% (Female 2), respectively. A Weibull function was fitted to the data to obtain the 75% and the 50% response threshold for both animals, i. e. the interpolated stimulus intensity to which the animals responded in 75% or 50% of the stimulus trials. 75% response thresholds were 20.8 mm s^−1^ (Male 1) and 6.2 mm s^−1^ (Female 1). 50% response thresholds were 9.4 mm s^−1^ (Male 1) and 0.4 mm s^−1^ (Female). The 50% threshold for Female 2, however, is influenced by one water velocity value (the value for the 5 cm water column) that could not be measured directly and was obtained from extrapolation. More conservatively, we assume a 50% threshold of 1.0 mm s^−1^ water velocity for Female 2, the velocity where the animal responded correctly in 52% of the stimulus trials.

Mean response latencies, defined as the time from the opening of the valve to the response of the animal, were between 2 and 3 s for Male 1 in all stimulus conditions (7–41 trials per stimulus condition), and between 1 and 2 s for Female 1 in all stimulus conditions (6–32 trials per stimulus condition).

## Discussion

### Sensitivity to horizontal water motions

The present study shows that the Australian water rat *H. chrysogaster* can sense, like the harbour seal *P. vitulina* (Dehnhardt et al. 1998a, 2001) and the California sea lion *Zalphus californianus* (Gläser et al. 2011), water motions with its vibrissae. Response thresholds with the hydrodynamic stimuli being detected on 50% of stimulus trials presented to *Hydromys* are only a few millimetres per second (in our experimental animals 9.4 mm s^−1^ and 1.0 mm s^−1^, respectively). A moving fish of 20 cm length may generate subsurface water motion amplitudes above 100 mm s^−1^ (e. g. Bleckmann et al. 1991; Nauen and Lauder 2002; Tytell and Lauder 2008; Lauder 2015). 80 mm s^−1^ have been measured in the wake of an accelerating 86 mm long fish, and 20 mm s^−1^ in a calmly swimming fish that had a total length of 86 mm (Hanke and Bleckmann 2004). Fish of this size are part of the prey spectrum of *Hydromys*. The present study reveals that the water motions they cause are much higher in amplitude than necessary to elicit a response in *Hydromys*. Woollard and McLean (1978) report that *Hydromys* were commonly observed eating goldfish of 12–14 cm in length, and estimate from fish scales found in the stomach and intestines of *Hydromys* that fish of up to 30 or 36 cm are consumed. The present study does not explicitly demonstrate that *Hydromys* uses prey-generated water motions for prey detection, however this conclusion is consistent with its vibrissal sensitivity and the sit-and-wait behaviour of the species.

Harbour seals (*Phoca vitulina*) detect and analyse subsurface water motions not only of the sinusoidally oscillating dipole type (Dehnhardt et al. 1998a), which bear some resemblance with the water movements generated by oscillating body parts, but also direct current water jets (Wieskotten et al. 2011; Niesterok et al. 2017a, b; Krüger et al. 2018). Direct current water jets occur, for example, in the tail wake of fishes (Hanke and Bleckmann 2004; Niesterok and Hanke 2013), where they often form the central part of vortex rings, and in the breathing currents of fishes (Niesterok et al. 2017b). As vortex rings produced by fish tails constitute strong and stable flow structures, they appear to be promising subjects for future studies on the discrimination and directional sensitivity of *Hydromys* to hydrodynamic stimuli, corresponding to studies on stationary harbour seals (Krüger et al. 2018).

### Sensitivity to surface waves

Surface waves generated by terrestrial insects trapped and struggling at the water surface and by aquatic animals touching the water surface from below, as well as surface waves generated by abiotic sources such as wind or falling objects, have been studied extensively by authors that were interested in predator–prey interactions (e. g. Lang 1980; Bleckmann 1985a, b; reviews by Bleckmann 1994 and Hanke 2020). Surface waves from struggling insects are broadband (typically 0–140 Hz) and low in amplitude, and originate from a more or less constant location for an extended time span in the order of seconds. Surface waves from aquatic animals that briefly touch the water surface have narrower frequency bands (typically up to 40 Hz for waves caused by vertebrates, up to 100 or rarely 140 Hz for waves caused by insects) and last for less than a second. Thus, the broadband stimuli used in experiment 1 in this study share key characteristics with the surface waves caused by terrestrial insects struggling at the water surface. The single-frequency surface waves used here resemble the surface waves caused by trapped insects only regarding their overall duration and their originating from a constant location, while they were somewhat artificial with regard to their frequency content. Broadband stimuli of 0–50 and 0–100 Hz used in this study resemble the surface waves caused by aquatic animals regarding their frequency content and their originating from a constant location, while they tend to exceed them in duration. It is remarkable that the water rats did not respond to a variety of surface wave stimuli that were offered and that included close approximations of surface waves generated by insects struggling at the water surface, which are potential prey items.

Contrary to surface feeding fish, aquatic amphibians, whirligig beetles, back swimmers, water striders, fishing spiders (for review see Bleckmann 1994) and crocodiles (Grap et al. 2015), the Australian water rats used in this study did not respond to pure surface waves, but exclusively to the complex hydrodynamic stimuli used in experiment 2 (stimuli 2). The amplitudes of both the sine waves and the broadband stimuli in experiment 1 (waves generated with a vibrating rod) were several orders of magnitude above the sensory thresholds of the surface feeding animals mentioned above. The fact that *Hydromys* never responded to pure surface waves may be due to a lack of sensitivity or a lack of motivation. We favour the first explanation, because considering the broad prey spectrum of *H. chrysogaster* it is unlikely that the surface waves applied here did not simulate attractive prey. This holds true at least for the large bandwidth stimuli whose frequency content came close to the frequency content of water surface waves generated by terrestrial insects struggling at the water surface (Bleckmann 1985a, b, 1988). An additional hypothesis to be tested in future studies is that *Hydromys* may be able to learn to detect pure surface waves (stimuli 1) as in experiment 1 after being trained with complex hydrodynamic stimuli (stimuli 2) as in experiment 2. However, if the water rats would respond to stimuli 1 after learning to respond to stimuli 2, we would still conclude that pure surface waves are probably not the adequate stimulus in the ecological context, as there was a striking contrast between not learning to respond to stimuli 1 for four weeks and spontaneously and strongly reacting to stimuli 2 in the very first trial.

The surface waves that were part of stimuli 2 at the 50% threshold are unlikely to be sufficient to elicit a response. If the 50% response threshold is expressed in terms of water column height by interpolation, we obtain 25.5 cm (animal 1) and 9.5 cm (animal 2), respectively. Surface wave amplitudes of the complex stimuli varied between 435 ± 143 µm (25 cm water column) and 65 ± 32 µm (15 cm water column). Thus, in animal 1, surface wave thresholds in experiment 2 were smaller than the pure surface waves used in experiment 1 by a factor of 2.1 (10 Hz sinus), and 1.6 (20 Hz sinus), albeit larger than the other pure surface waves applied. For animal 2, the surface waves at the behavioural threshold in experiment 2 were smaller than the surface waves in experiment 1 by a factor of 1.8–14, and thus unlikely to elicit a response alone. It is still possible, however, that the surface waves included in hydrodynamic stimuli 2 may have aided in the detection process.

### Ecological implications

This pilot study presents results from three (experiment 1) or two (experiment 2) individuals of *H. chrysogaster* that were available at the time of the experiments. The limited number of experimental animals does not allow for a survey of intraspecific variation. However, the three (or two, respectively) individuals were apparently normal and healthy representatives of the species and most probably provide typical examples of the sensory abilities of *Hydromys*; superior abilities in free-ranging conspecifics with extensive experience in the natural habitat cannot be excluded.

Fish, aquatic amphibians, crocodilians, crustaceans, cephalopods, and many semiaquatic insects and fishing spiders (Bleckmann 1994; Soares 2002; Marshall et al. 2014) extract information about their environment from prey-generated hydrodynamic stimuli. Among mammals, harbour seals (*Phoca vitulina*) not only detect water movements with their vibrissae (Dehnhardt et al. 1998a), but also use vibrissal information to follow the hydrodynamic trails caused by moving objects (Dehnhardt et al. 2001) over a distance where vision is useless (Weiffen et al. 2006). The present study provides the first evidence that a rodent can detect hydrodynamic stimuli. Its hydrodynamic sensitivity is sufficient to sense a fish swimming by, but probably not to detect an insect struggling at the water surface. Most likely the water rat’s hydrodynamic perception aims at relatively large prey.

The visual system of *Hydromys* is adapted to a nocturnal life style. *Hydromys* is short-sighted and lacks adaptations for underwater vision (M. Schleef and G. Dehnhardt, unpublished). Perception of movement appears to be good (Woollard et al. 1978). This makes it conceivable that prey items, or surface waves caused by prey items, may be perceived visually in bright nights or during daytime while *Hydromys* is in lurking position, as the sit-and-wait behaviour of *Hydromys* is not exclusively nocturnal (own observations).

Other senses of *Hydromys* have not been studied. The outer ear of this water rat is not remarkably different from that of land-living rodents, making it likely that its sense of hearing is especially well developed. Observations in our laboratory suggest that olfactory prey perception may also play a significant role, at least as long as the water rat’s head is not submerged. Specifically, the odour of fresh fish in air, which was not part of the regular diet, could induce excited sniffing with the nose raised.

Detection of fluid flow, specifically air flow, by a rodent using its vibrissae has also been reported by Yu et al. (2016, 2019) for rats (*R. norvegicus*). Rats are able to respond to air flow and perform anemotaxis, i.e. orient towards the wind direction. This behaviour should be suited to track down a food source whose odour is carried away by the wind. Similar abilities are conceivable in *Hydromys* as well.

Considering the natural sit-and-wait behaviour of *H. chrysogaster* (Fig. 1a), the results of our study strongly support the hypothesis that the perception of prey-generated water movements plays a role in foraging in the Australian water rat. Subsurface water motions generated by fish can exceed the water rat's detection thresholds greatly. Fish-generated surface waves may add to the detection process.

Fishing spiders of the genus *Dolomedes* as well as semiaquatic insects of the genus *Notonecta* also attempt to catch small fish passing by (Bleckmann and Lotz 1987; Mail et al. 2018). In these predators, fish catching behaviour is triggered by the surface waves and subsurface water motions caused by fish, but also by any direct incidental contact with a fish. Most likely incidental vibrissal contact with a fish will also trigger prey capture behaviour in *Hydromys*. However, sensing of hydrodynamic stimuli will significantly extend the detection range of the vibrissal system. To what accuracy the direction and distance of the prey can be localized remains to be investigated. Further important follow-up questions include how Australian water rats may cope with the hydrodynamic noise pervasive in running waters.

## Electronic supplementary material

Below is the link to the electronic supplementary material.

Supplementary file1 (PDF 666 kb)

Supplementary file2 (AVI 10482 kb)
