# Supplementary material for: Hydrodynamic reception in the Australian water rat, Hydromys chrysogaster
Source: J Comp Physiol A Neuroethol Sens Neural Behav Physiol. 2020 Apr 18;206(4):517–26. doi: 10.1007/s00359-020-01416-8 (PMC7314732; doi:10.1007/s00359-020-01416-8)
Supplement: Supplementary file 1 — Supplementary file1 (PDF 666 kb) [file 359_2020_1416_MOESM1_ESM.pdf]

W. Hanke, S. Meyer, H. Bleckmann, G. Dehnhardt
Hydrodynamic perception in the Australian water rat, *Hydromys chrysogaster*

Supplementary Materials: Methods

Floating particle recording and evaluation

Videos of floating particles, illuminated by a laser light sheet 5 mm below the water surface, were recorded with an S-VHS recorder (see materials and methods section) at the time of the experiments. Videos were digitized post measurement using an LG V4745 video recorder whose signal was looped through a JVC SR-VS20 video recorder to obtain a Digital Video (DV) signal. The DV signal was read into a computer via IEEE 1394 (Firewire®). Not only the particles in the laser light sheet, but also particles floating on the water surface were illuminated, the latter by reflected laser light with lower intensity. While this effect prevented the videos from being evaluated with correlation techniques as they are common in particle image velocimetry (PIV), it also offered the opportunity to measure water velocities in two different planes, namely the laser light sheet 5 mm below the water surface as well as the water surface itself.

Water velocities were measured by manually marking the positions of identical particles or particle clusters in successive video frames using ImageJ 1.52a (http://imagej/nih.gov/ij). Particle displacement was then divided by the time between frames to obtain particle velocity, and thus water velocity. Temporal resolution was increased to 50 frames per second by separating the interlaced video frames into half frames using MatLab where necessary.

Velocity at the water surface was measured in nine equidistant positions (positions 1-9, see Fig. S1) at least every 0.2 s, or at shorter time intervals where required. Velocity in the laser light sheet was measured in three positions along the edge of the image close to the stimulus generator (positions L1-L3, see Fig. S1). Exact positions of the particles used for measurements could deviate from the defined measurement postions 1-9 by at most 2 mm, depending on particle availability, while positions L1-L3 were adjusted to include the visually identified fastest particles. One stimulus presentation per water column height was chosen for this detailed evaluation. The time course of these water velocities is exemplified in Fig. S2.

Velocities at the surface were more uniform in direction than subsurface water motions, where vortices occured. Surface velocities are also the relevant velocities near the behavioural thresholds of the experimental animals. Before the stimulus, a uniform background flow was present in the experimental pool that was very slow, but not quite negligible. For surface velocities, therefore, the difference vectors between the velocities during stimulus presentation and the velocity before the stimulus were taken as the stimulus-induced velocities. In the light sheet 5 mm below the surface, however, water velocities exceeded the background flow greatly (as far as the stimulus-induced flow reached the measurement area during the 4 s response window) so that background flow could be neglected.

Based on the identified flow patterns and on additional visual screening, maximal velocity was measured by marking individual particles in four to five stimulus presentations per water column height. Means of water velocity and water acceleration from four to five stimulus presentations per water column height, along with the standard deviation, are given in Table 2.

Supplementrary Fig. S1

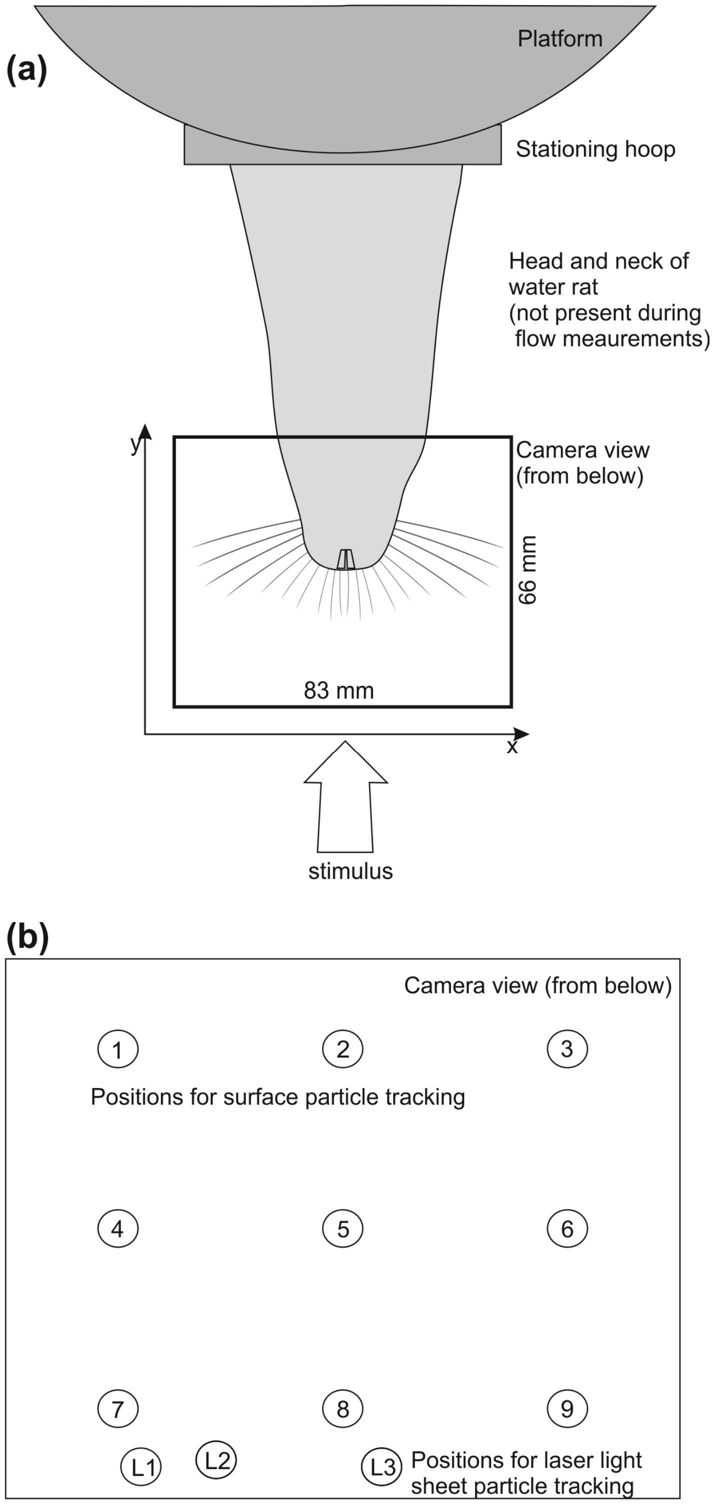

Fig. S1 Camera field of view for flow measurements. **a** Position of field of view in front of the experimental platform. A horizontal laser light sheet is filmed from below with a submerged camera. **b** The velocity of particles floating on the water surface was measured in positions 1 - 9. The velocity of particles in the light sheet 5 mm below the surface was measured in positions L1 - L3 along the edge of the field of view closest to the stimulus generator; positions L1 - L3 were adapted along the x axis to include the fastest particles.

Supplementrary Fig. S2

(a) water column height 40 cm

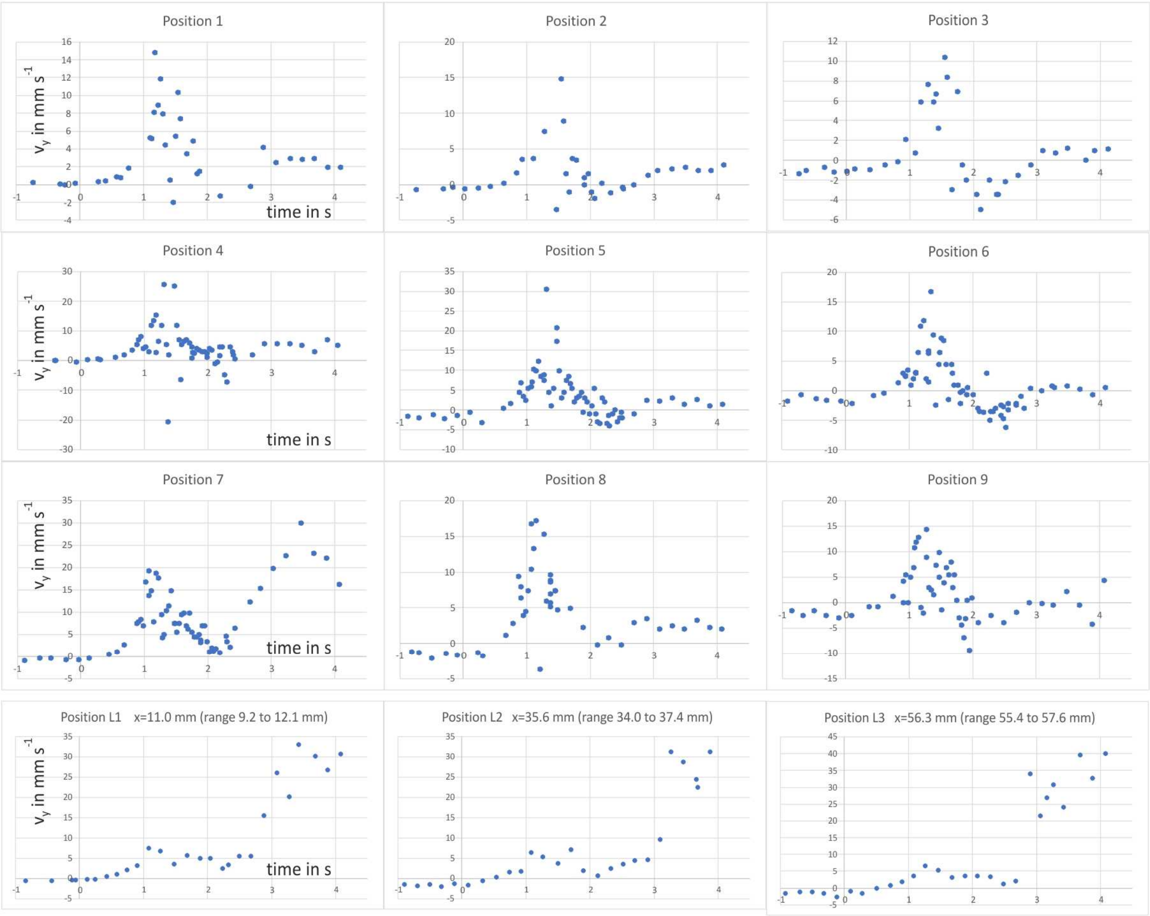

(b) water column height 25 cm

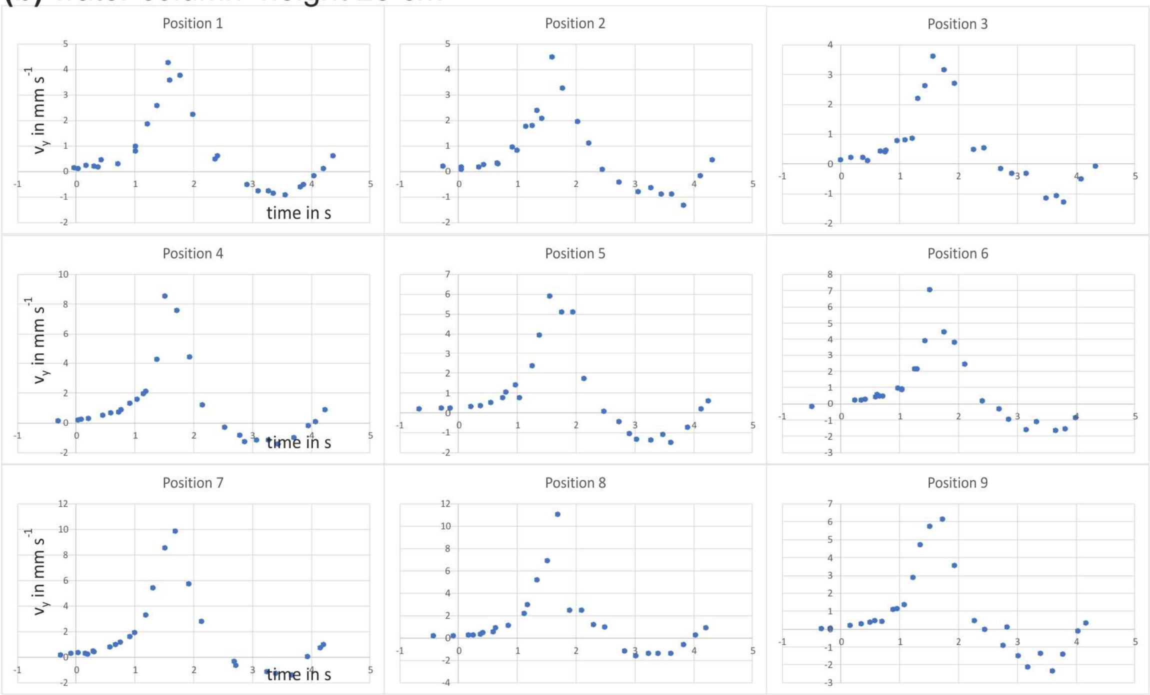

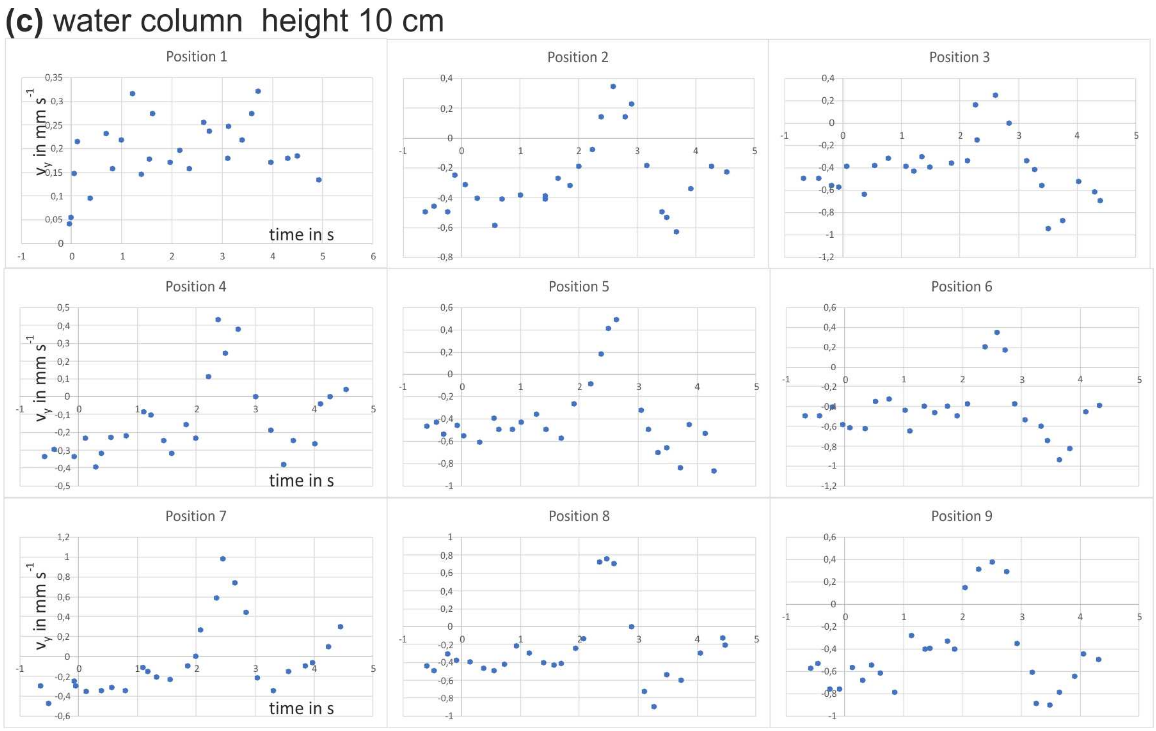

Fig. S2 Examples of water velocities over time measured in Positions 1-9 and L1-L3 (cf. Fig. S1). Depicted here is the y component of the velocity. **a** Stimulus with 40 cm water column height, the strongest stimulus used during threshold assessment. **b** 25 cm water column, the stimulus close to the 50% threshold of animal M1. **c** 10 cm water column, the stimulus close to the 50% threshold of animal F1. Water flow in the laser light sheet reached the field of view only in **a**.

Supplementary Movie 1

Supplementary movie 1 presents examples of flow recordings from one stimulus presentation per water column height. The first two examples are slowed down by a factor of two. The two stimuli closest to the 50% thresholds of the two animals (but slightly stronger than the calculated 50 % threshold stimuli) are pointed out. Note the particles moving in two different layers, namely the laser light sheet in the foreground and the water surface in the background. Overlaid text in the upper left corner resulted from the operation of the recorders and is irrelevant. The onset of the LED light in the center indicates the operation of the valve that triggered the stimulus.
